# Table of Two Hundred and Twenty Cases of Fracture

**Published:** 1842-10-29

**Authors:** 


In the 11th Part of the Cyclopedia of Practical Surgery, there is the following table of two hundred and twenty cases of Fracture admitted into Guy's Hospital in one year:

| FRACTURES OF THE | | Nos. | | Reported, open, or compound. |
|---|---|---|---|---|
| Femur | | | 48 | |
| Tibia and fibula | | 36 | | |
| *Tibia* | | 13 | | |
| *Fibula* | | 13 | | |
| Leg | | 4 | 66 | 10 |
| Humerus | | | 30 | 2 |
| Fingers | | | 18 | 18 |
| Ribs | | | 11 | emphysema. 4 |
| Patella | | | 8 | |
| Radius | | 4 | | |
| Ulna | | 1 | | |
| Olecranon | | 2 | | |
| Radius and Ulna | | 4 | 11 | 1 |
| Clavicle | | | 5 | |
| Skull | | | 4 | |
| Ossa Nasi | | | 3 | 3? |
| Lower Jaw | | | 3 | 3? |
| | | | 1 | |
| Great Toe | 2 | | | |
| Astragalus | 1 | | 4 | 3 |
| Spine | 1 | | | |
| Of bones, not mentioned, of difficult diagnosis, as about great joints, &c. | | | 8 | 1 |
| | | | 220 | |

